# A newly developed strain of *Enterococcus faecium* isolated from fresh dairy products to be used as a probiotic in lactating Holstein cows

**DOI:** 10.3389/fvets.2022.989606

**Published:** 2022-10-13

**Authors:** Hossam H. Azzaz, Ahmed E. Kholif, Hussein A. Murad, Einar Vargas-Bello-Pérez

**Affiliations:** ^1^Dairy Science Department, National Research Centre, Giza, Egypt; ^2^Department of Animal Sciences, School of Agriculture, Policy and Development, University of Reading, Reading, United Kingdom

**Keywords:** *Enterococcus faecium*, digestion, feed utilization, lactic acid bacteria, milk production

## Abstract

The objective of this study was to determine the ability of an isolated strain (EGY_NRC1) or commercial (NCIMB 11181) *Enterococcus faecium* as a probiotic for lactating cows. Two experiments were conducted: In Experiment 1, the effects of three levels (1, 2, and 3 g/kg diet, DM basis) of isolated and commercial *E. faecium* on *in vitro* ruminal fermentation kinetics, gas, methane (CH_4_) and nutrient degradability were determined. In Experiment 2, thirty multiparous Holstein cows (633 ± 25.4 kg body weight) with 7 days in milk, were randomly assigned to 3 treatments in a completely randomized design in a 60-day experiment. Cows were fed without any additives (control treatment) or supplemented with 2 g/kg feed daily of *E. faecium* EGY_NRC1 (contain 1.1 × 10^9^ CFU/g) or commercial *E. faecium* NCIMB 11181 (contain 2 × 10^12^ CFU/g). Diets were prepared to meet cow's nutrient requirements according to NRC recommendations. Probiotic doses were based on the *in vitro* Experiment 1. Feed intake, digestibility, blood parameters and lactation performance were evaluated. In Experiment 1, the isolated *E. faecium* linearly and quadratically increased (*P* < 0.001) *in vitro* total gas production (TGP), the degradability of dry matter (dDM) and organic matter (dOM) while decreased (*P* < 0.05) methane (CH_4_) percent of TGP, NH_3_CH_4_ production, and pH. The commercial *E. faecium* increased TGP and decreased (*P* < 0.01) CH_4_ production, pH and increased the dDM and dOM, short chain fatty acids and ruminal NH_3_-N concentration. In Experiment 2, the isolated *E. faecium* increased (*P* < 0.01) total tract digestibility of DM, neutral and acid detergent fiber, daily milk production and feed efficiency compared to the control treatment without affecting feed intake and milk composition. Moreover, the isolated *E. faecium* increased (*P* < 0.05) the proportion of C18:1 *trans*-9, C18:2 *cis*-9-12 and C18:2 *trans*-10 *cis*-12. Both isolated and commercial *E. faecium* improved (*P* < 0.01) organic matter, crude protein and nonstructural carbohydrates digestibility, increased serum glucose (*P* = 0.002) and decreased serum cholesterol (*P* = 0.002). Additionally, both *E. faecium* strains decreased C23:0 (*P* = 0.005) in milk. In conclusion, the use of *E. faecium* (isolated and commercial) at 2 g/kg DM of feed improved feed efficiency and production performance, with superior effects on animal performance from isolated *E. faecium* compared to the commercial one.

## Introduction

Probiotics and prebiotics have been administered to animals for several years to enhance their health and production. In animal production, probiotics are now widely accepted as safe and sustainable alternatives to antibiotics (1, 2). For many years, lactic acid bacteria (LAB) have been used in livestock production, as probiotic supplements or as silage preservative by inhibiting pathogenic microorganisms (e.g., fungal and clostridial growth) and increasing lactic acid formation (3, 4). Normally, LAB are predominantly found in the gastrointestinal tract of animals and humans and are also found in dairy products (5).

In livestock, different bacterial and fungal species (i.e., *Bacillus, Bifidobacterium, Enterococcus, Lactobacillus, Propionibacterium, Megasphaera elsdenii* and *Prevotella bryantii*) have been used as probiotics as well as strains of *Aspergillus* and *Saccharomyces* (1, 6). Strains of LAB including *Lactobacillus, Bifidobacterium* and *Streptococcus* are commonly used as probiotics in ruminant feed (1, 7). LAB can alter ruminal fermentation and enhance nutrient digestibility and productive performance (8). LAB reduce oxygen from the rumen environment and prevent excess of ruminal lactate production, inhibit ruminal pathogens, and modulate the microbial balance (9). Previous studies have reported the effect of LAB in ruminant diets, including the increased yield of microbial biomass (10), reduced methane (11) and increased dry matter (DM) digestibility (12). Recently, it has been reported that feeding dairy cows with *L. casei* TH14 can improve feed utilization, rumen fermentation parameters and milk production (13).

The use of commercial LAB can increase total mixed rations costs and finding alternatives that keep similar efficiency to that from commercial products is of great interest for production improvement. Also, research on novel strains of microorganisms with different origins and properties is still needed. Therefore, the objective of this study was to determine the ability of an isolated strain (EGY_NRC1) or commercial (NCIMB 11181) *Enterococcus faecium* as a probiotic for lactating cows. The hypothesis of this study was that the in-feed supplementation of commercial or isolated *Enterococcus faecium* would lead to similar effects on feed utilization, milk production, milk composition and milk fatty acid profile in early lactating dairy Holstein cows.

## Materials and methods

### Isolation and identification of *E. faecium*

Lactic acid bacterial strains were isolated from 15 samples of fresh dairy products (homemade 8 samples of yogurt and 5 samples soft white cheese). For each sample, 10 g were added to 90 ml sterile saline solution and homogenized by vortex for 10 min. Decimal dilutions were placed on double layered M17 agar plates then incubated for 48 h at 30°C. Well defined round colonies were selected randomly and only Gram-positive catalase-negative cocci were retained and stored in M17 broth for further experiments.

**Figure 1:**
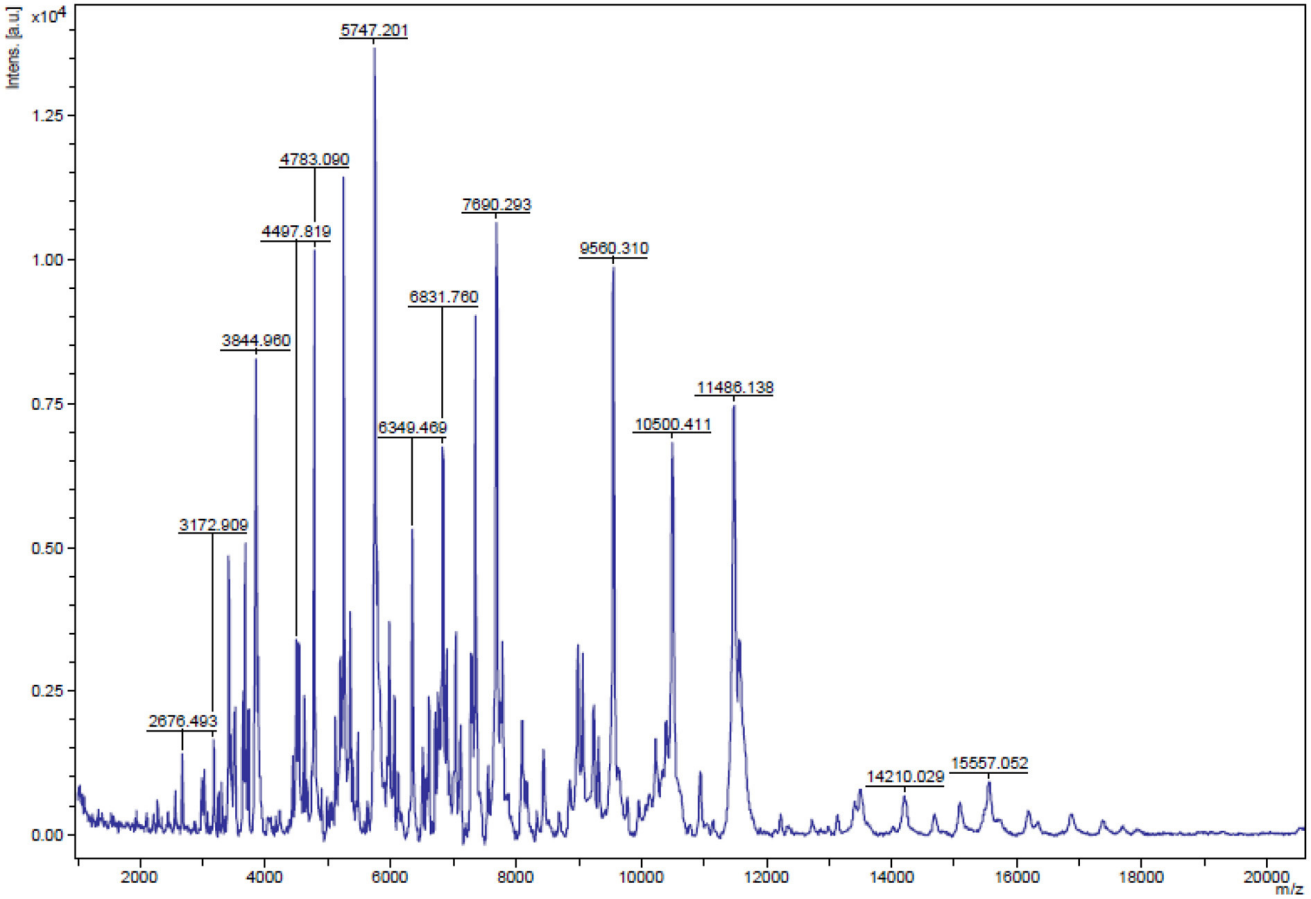
Result of the matrix-assisted laser desorption ionization time-of-flight mass spectrometry (MALDI-TOF MS) analysis. The MALDI-TOF MS showed that the isolated strain of *E. faecium* that possess antimicrobial activity was initially identified with a high confidence value of 2.31 that indicated a reliable identification of the isolate up to species level.

To characterize the selected isolate, the carbohydrate fermentation pattern (14) of the selected isolate (that possessed antibacterial activity) and its ability to produce ammonia (NH_3_) from arginine (15) was examined. After that, the strain was identified by matrix-assisted laser desorption ionization-time-of-flight mass spectrometry (MALDI-TOF MS) and 16S ribosomal DNA (rDNA) sequencing. The isolate was identified (Figure 1) *via* the analysis of its total proteome in which a score with more than 1.7 indicates genus identification and a score with more than 2 is the confidence value at the species level (16).

For DNA extraction, genomic DNA was used from the selected isolate that was cultivated at 37°C for 24 h. Freshly prepared culture was subjected to16S rDNA PCR partial amplification by use of Qiagene genomic DNA purification kit. The genomic DNA was used as a template for PCR amplification of a segment of its 16S rRNA gene. The two primers used were previously described by Liu et al. (17), 8f (5_AGAGTTTGATCCTGGCTCAG-3) and 1495 R (5_CTACGGCTACCTTGTTACGA-3). The PCR products yielded were analyzed on a 1% (w/v) agarose gel after staining with ethidium bromide. The PCR products were separated on an agarose gel, followed by ethidium bromide staining to check for the presence of a unique amplicon. When a gene from a particular species was amplified using a primer initially designed for a different species, the corresponding amplicon was purified by Promega Wizard Genomic DNA Purification Kit and sequenced by HVD life science, Germany.

For phylogenetic analysis (data not shown), the 16S rDNA sequence obtained was added to publicly available bacterial 16srRNA sequences and integrated into the database with the automatic alignment tool. Phylogenetic analysis was inferred using neighbor joining method and the phylogenetic tree was constructed (18). The isolated LAB was identified as *E. faecium* EGY_NRC1 with NCBI accession number MW856655.

The biomass production of the isolated LAB was done on whey permeate media with the following composition (/L permeate): 5 g yeast extract, 5 g peptone, 0.5 g magnesium sulfate, 3 g ammonium chloride and 2.5 g ascorbic acid. The medium was inoculated individually with 5% (v/v) of the isolated LAB culture (24 h old age activated M17 broth culture) and then incubated at 37°C for 48 h. The cultured biomasses were separated by centrifugation at 6,000 rpm for 15 min at 4°C then added to dry permeate as a carrier agent, and dried in oven at 37°C. The un-inoculated media were used as control. Unpublished data showed that the viable count of the strain after pre-incubation at 60°C for 30 min was enumerated and the result indicated that the remaining count was 3.274 log CFU/mL in compared to 5.477 log CFU/mL without pre-incubation. The reported viable count was determined after drying.

### Experiment 1

#### *In vitro* evaluation

Using a stomach tube, rumen liquor was obtained from three adult Barki sheep (51 ± 2.6 kg of body weight) fed a fixed amount of concentrate (500 g) and *ad libitum* berseem hay daily. The rumen contents (liquid and solid contents 1:1 v/v) were collected before morning feeding, kept in pre-warmed thermo containers at 39°C under anaerobic conditions. About 500 mL of ruminal fluid was collected from all ewes of each treatment. To avoid saliva contamination, the first 50 mL of the rumen fluid samples were discarded. The rumen fluid was mixed for 10 s, squeezed through four layers of cheese cloth, and maintained in a water bath at 39°C under continuous CO_2_ flushing until inoculation (19). Three incubation runs were performed in three different weeks. Rumen contents obtained from the three sheep were combined for each run. Animal use for this trial was approved by the technical committee of the Science, Technology & Innovation Funding Authority (STDF), Egypt (project STDF 33413).

A total mixed ration composed of (per kg DM) 300 g berseem clover, 300 g corn silage, 150 g soybean meal, and 250 g yellow corn was used as a substrate. The nutrient contents of feed ingredients and basal diet are shown in Table 1. The *in vitro* total gas production (TGP) assay was conducted as described by Theodorou et al. (19) and adapted to the semi-automatic system of Mauricio et al. (20). Ground substrate samples (500 mg of DM) were incubated in 120 mL serum bottles (5 bottles per dose of LAB at each time). LAB (isolated and commercial) was included at 0, 1, 2, and 3 g/kg DM substrate. The isolated bacteria contained 1.1 × 10^9^ CFU/g. Levels of inclusion were based on initial screening of many doses of *E. faecium* on ruminal fermentation (2, 21). After dispensing, bottles were closed with rubber stoppers, shaken manually, and incubated at 39°C in a forced-air oven for 48 h. The bottles were shaken at 1 h intervals during incubation. The amount of TGP was calculated according to the regression equation {V = 4.974 12 × p + 0.171; *n* = 500, *r*^2^ = 0.98; [V is gas volume (mL); p is measured pressure (psi)]} obtained in our laboratory under our conditions according to the gas volume vs. pressure. Bottles containing only buffered rumen fluid without substrate were considered as blanks. At each incubation time, 5 mL of headspace gas was taken from each bottle and infused into a Gas-Pro detector (Gas Analyzer CROWCON Model Tetra3, Abingdon, UK) to measure the concentration of CH_4_ and carbon dioxide. The control and experimental treatments were tested in 6 bottles (analytical replicates) and three incubation runs in three consecutive weeks with 3 bottles containing inoculum and buffer but no feed (blanks).

**Table 1:** Chemical composition of ingredients and control diet used in the *in vitro* and *in vivo* experiments (g/kg of dry matter).

	**Yellow corn^a^**	**Soybean meal^a^**	**Corn silage^a^**	**Berseem clover^a^**	**Basal diet^b^**
Dry matter, wet weight	896	898	891	889	893
Organic matter	985	930	915	870	921
Crude protein	83	422	77	170	158
Ether extract	51	48	21	32	36
Ash	15	70	85	130	79
Non-structural carbohydrates^c^	666	313	286	258	377
Neutral detergent fiber	185	147	531	410	351
Acid detergent fiber	37	65	320	270	196

^*a*^Analyzed values. ^*b*^Calculated values. Used as a diet for all treatments. ^*c*^Non-structural carbohydrates = 1,000–(neutral detergent fiber + crude protein + ether extract + ash).

At 48 h (the end of incubation), fermentation was terminated by immersing the bottles in ice. For each LAB level, 3 bottles were used to measure the pH, NH_3_-N (22) and short chain fatty acids (SCFA) by steam distillation and titration (23), whereas the other 3 bottles were filtered in pre-weighed crucibles and washed with hot water then acetone, and the residual DM and ash were estimated to determine true DM, organic matter (OM), neutral detergent fiber (NDF) and acid detergent fiber (ADF) degradability (dDM, dOM, dNDF, and dADF, respectively).

### Experiment 2

#### Animals

Cows were managed in accordance with the Guide for the Care and Use of Agricultural Animals in Agricultural Research and Teaching, 3^rd^ edition, 2010 (Federation of Animal Science Societies, Champaign, IL, USA). The protocol was approved by the technical committee of the Science, Technology & Innovation Funding Authority (STDF), Egypt (project STDF 33413).

During the first week of lactation, thirty lactating Holstein cows (633 ± 25.4 kg body weight, 3 ± 1 parity, 7 ± 1 days in milk, with a previous milk production of 24 ± 2.2 kg/d, were assigned randomly to one of three experimental treatments in a complete randomized designed with 10 cows per treatment for 60 days.

Cows were divided into three barns in soil-surfaced free stalls (122 × 175 cm^2^/cows), under shade, without any bedding and with free access to water. Cows were fed *ad libitum* a diet containing [per kg DM] 300 g berseem clover, 300 g corn silage, 150 g soybean meal and 250 g grounded corn grain, to meet their nutrient requirements according to NRC (24) recommendations for 650 kg cow with 20 kg DM intake and 35 kg daily milk production. Animals were fed 10% more of the expected dry matter intake to ensure collection of orts. The diet fed to cows was the same for the *in vitro* experiment.

Cows were fed their diets without any additives (control treatment) or supplemented with 2 g/kg feed daily of *E. faecium* EGY_NRC1 (isolated to contain 1.1 × 10^9^ CFU/g) or commercial *E. faecium* NCIMB 11181 (isolated to contain 2 × 10^12^ CFU/g; ADM Protexin Limited, Lopen Head, Somerset, TA13 5JH UK). The doses of probiotics were based on the results obtained from Experiment 1 (*in vitro* experiment). Diets were offered twice a day at 08:00 and 16:00 h. The additives were mixed with all feedstuffs using a feeding wagon. Before use, probiotics were kept at 4°C. Samples of feed ingredients were taken daily, composited weekly, dried at 60°C in a forced-air oven for 48 h (method 930.15) (22) and stored for chemical analyses.

The total mixed ration was prepared and distributed using a horizontal mixer system (DeLaval, Ontario, Canada) after mixing for 20 min. Samples of feed were taken daily, composited weekly, dried at 60°C in a forced-air oven for 48 h (22) and stored for chemical analysis. The nutrient contents of the feed ingredients are shown in Table 1. Cows were weighed on a digital multi-purpose platform scale at the beginning and at the end of the experiment.

#### Nutrient intake and digestibility

Feed intake was recorded daily by weighing the total daily amount of feed offered to each cow and the total daily amounts of weighed orts. On the 4^th^ and 8^th^ week, nutrient digestibility trials were conducted, in which acid insoluble ash was used as an internal indigestibility marker (25). Acid-insoluble ash contents of feeds and feces were determined gravimetrically after drying, burning, boiling of ash in hydrochloric acid, filtering and washing of the hot hydrolysate, and re-burning. Coefficients of digestion were calculated according to Ferret et al. (26). For the digestibility trial, fecal samples were collected from all cows twice daily at 09:00 and 16:00 h, dried at 60°C for 48 h in a forced-air oven and pooled by cow. The nutritive value of diets expressed as total digestible nutrients (TDN), digestible energy (DE), metabolizable energy (ME), net energy for lactation (NEL) were calculated according to NRC (24) equation, while the net energy requirements for lactation equivalent of 1 kg of standard air-dry barley (UFL) was calculated according to INRA (27) equation.

The dried feed, orts and fecal samples were ground to pass a 1-mm screen using a Wiley mill and analyzed for DM (method ID 930.15), ash (method ID 942.05), nitrogen (method ID 954.01) and ether extract (EE; method ID 920.39) according to the AOAC (22) official methods. Neutral detergent fiber was determined by the procedure of Van Soest et al. (28) with the use of alpha amylase and sodium sulphite. Acid detergent fiber was analyzed according to the AOAC (22). Lignin was analyzed according to Van Soest et al. (28).

#### Sampling and analysis of blood serum

On the last day of the 4^th^ and 8^th^ weeks of the experiment, individual blood samples (10 mL) were taken before morning feeding at 08:00 h from the jugular vein. Blood samples were centrifuged at 4,000 ×*g* for 20 min at 4°C. The serum was separated into 2 mL Eppendorf tubes and frozen at −20°C until analysis. By using specific kits (Spinreact, Ctra. Santa Coloma, Girona, Spain) and following the manufacturers' instructions, blood serum samples were analyzed for total protein, albumin, globulin, urea-N, glucose, cholesterol, triglycerides, aspartate aminotransferase (AST), and alanine aminotransferase (ALT). The globulin concentration was calculated by subtracting the albumin values from their corresponding total protein values.

#### Milk sampling, and milk composition

Cows were milked (DeLaval parallel parlor P2100, SE-147 21 Tumba, Sweden) three times daily at 04:00, 12:00 and 20:00 h, and individual milk samples (30 g/kg of milk yield) were collected at each milking. A mixed sample of milk (proportional to amounts isolated in each milking time) was taken daily every 2 weeks to determine milk composition. Milk samples were analyzed using infrared spectrophotometry (Ekomilk-M ultrasonic milk analyzer, EON Trading 2000, INC, Bulgaria).

The gross energy content was calculated according to Tyrrell and Reid (29). The milk energy output (MJ/d) was calculated as milk energy (MJ/kg) × milk yield (kg/d). The energy corrected milk (ECM) and 4% fat corrected milk (FCM) were calculated according to Sjaunja et al. (30) and Tyrrell and Reid (29), respectively.

### Statistical analyses

Data from *in vitro* measurements were analyzed using the GLM procedure of SAS (SAS Inst. Inc. Cary, NC, USA) in a completely randomized design using the following model: Y_ij_ = μ + D_i_ + E_ij_, where Y_ij_ represents the measured variable, μ is the overall mean, D_i_ is the LAB dose, and E_ij_ is the experimental error. Data from each of the three runs within the same sample were averaged prior to the statistical analysis. Polynomial (linear and quadratic) contrasts were used to examine dose responses for increasing levels of LAB.

Data from *in vivo* measurements were analyzed as a completely randomized design with repeated measures using the PROC MIXED procedure of SAS (SAS Institute, Cary, NC, USA), considering sampling time as repeated measures and individual cow as the experimental unit.

Data for variables measured daily for each week were averaged before statistical analyses. The statistical model included the treatment effect, week effect and the treatment × week interaction. Animal nested within treatment was considered the random effect, while treatment was the fixed effect. Two covariance structures were considered in the REPEATED statement in PROC MIXED: compound symmetry (cs) and auto-regressive [AR(1)]. The error structure, with the lowest Akaike information criteria, that fits the statistics was selected for the model. When the *F*-test was significant at *P* < 0.05, means were compared by applying the probability of difference option of the least squares means statement. Significance was declared at *P* < 0.05.

## Results

### Experiment 1 (*in vitro* experiment)

Results of TGP and CH_4_ proportions differed between the isolated and commercial strains of *E. faecium* (Table 2). The inclusion of isolated *E. faecium* linearly and quadratically increased (P < 0.001) TGP, while linearly and quadratically decreased (*P* < 0.05) proportional CH_4_, CH_4_ production (per g dDM, g dOM, g dNDF, g dADF) and pH, and increased SCFA (linear and quadratic effects, *P* < 0.001). Linear increases in dDM and dOM were observed with the isolated *E. faecium*, with no effect on dNDF or dADF. The inclusion of isolated *E. faecium* did not affect the concentration of ruminal NH_3_-N.

**Table 2:** *In vitro* fermentation from Experiment 1 (mean values), where a basal diet was supplemented with isolated or commercial *E. faecium* as probiotics at 1, 2 or 3 g/kg DM.

	**Control**	**Isolated probiotics** ^**a**^			**Commercial probiotics** ^**a**^			**Pooled SEM**	**Isolated probiotics**		**Commercial probiotics**		**Control vs. others**	**Isolated vs. Commercial**
		**1**	**2**	**3**	**1**	**2**	**3**		**Linear**	**Quadratic**	**Linear**	**Quadratic**		
TGP, mL/g DM	114	122	126	121	123	115	129	1.1	< 0.001	< 0.001	0.066	0.090	< 0.001	0.001
CH_4_, %	25.1	20.4	18.5	19.9	20.3	21.2	19.9	0.36	< 0.001	< 0.001	< 0.001	< 0.001	< 0.001	0.005
CH_4_/g dDM	59.7	47.9	42.9	45.5	47.7	42.8	45.0	1.26	< 0.001	< 0.001	< 0.001	< 0.001	< 0.001	0.813
CH_4_/g dOM	52.3	42.5	38.2	40.4	42.6	39.9	40.5	1.05	< 0.001	< 0.001	< 0.001	< 0.001	< 0.001	0.473
CH_4_/g dNDF	79.9	62.5	55.0	58.7	65.8	60.6	61.9	3.53	< 0.001	0.005	0.007	0.036	< 0.001	0.173
CH_4_/g dADF	112	86	74	80	91	83	88	7.1	0.002	0.032	0.016	0.080	0.008	0.244
dDM, g/kg	482	519	546	532	521	569	528	13.2	0.006	0.061	0.003	0.004	0.006	0.505
dOM, g/kg	549	585	611	597	583	613	586	14.0	0.010	0.086	0.032	0.040	0.004	0.726
dNDF, g/kg	365	401	427	413	379	407	399	20.4	0.072	0.235	0.162	0.591	0.084	0.280
dADF, g/kg	266	292	318	304	278	297	290	19.5	0.119	0.321	0.308	0.633	0.160	0.321
pH	6.65	6.38	6.33	6.35	6.35	6.30	6.33	0.038	< 0.001	0.006	< 0.001	< 0.001	< 0.001	0.377
SCFA, mmol/L	1.18	1.37	1.45	1.45	1.38	1.41	1.41	0.036	< 0.001	0.010	< 0.001	0.006	< 0.001	0.412
NH_3_-N, mg/dL	12.7	13.1	13.2	13.1	13.0	13.2	13.1	0.18	0.064	0.199	0.111	0.235	0.031	0.710

dAFD, acid detergent fiber degradability; CH_4_, methane; dDM, dry matter degradability; dNDF, neutral detergent fiber degradability; NH_3_-N, Ammonia-N (mg/dL); dOM, organic matter degradability (g/kg); SCFA, short chain fatty acids (mmol/L); TGP, *in vitro* total gas production (mL/g DM). ^*a*^The isolated product contained *E. faecium* EGY_NRC1 registered in the NCBI with accession number MW856655 and contained 1.1 × 10^9^ CFU/g product evaluated at 1, 2 or 3 g/kg DM. ^*b*^The isolated product contained *E. faecium* NCIMB 11181 with a total viable count 2 × 10^12^ CFU/g (ADM Protexin Limited, Lopen Head, Somerset, TA13 5JH UK) evaluated at 1, 2 or 3 g/kg DM.

Increased TGP and decreased proportional CH_4_ (linear and quadratic effects, *P* < 0.01) were observed with the inclusion of the commercial *E. faecium* strain; however, CH_4_ production (per g dDM, g dOM, g dNDF, g dADF) and pH linearly decreased (*P* < 0.01), and dDM, dOM, SCFA and ruminal NH_3_-N concentration increased, without affecting dNDF and dADF.

### Experiment 2 (lactation experiment)

#### Feed intake, nutrient digestibility, and blood measurements

Feeding *E. faecium* diets did not affect total feed intake (Table 3). Compared to the control, the highest (*P* < 0.01) DM, NDF and ADF degradabilities were observed with the isolated *E. faecium* followed by the commercial *E. faecium*, while both isolated and commercial *E. faecium* improved (*P* < 0.01) OM, CP, EE and NSC digestibility. The isolated *E. faecium* followed by the commercial *E. faecium* showed higher (*P* < 0.01) diet's nutritive value calculated as TDN, DE, ME, NEL, and UFL compared to the control treatment. Both the probiotic strains influenced an increased (*P* < 0.05) intake of digestible OM, TDN, ME and digestible CP compared to control, while between the strains the improvement in TDN, ME intake was superior (*P* < 0.05) in isolated than the commercial strain.

**Table 3:** Experiment 2: Intake, nutrient digestibility and nutritive value of diet supplemented with isolated and commercial *E. faecium* as probiotics and fed to lactating Holstein cows.

	**Diet** ^**a**^			**SEM**	***P*** **-value**		
	**Control**	**Isolated**	**Commercial**		**Diet**	**Control vs. others**	**Isolated vs. Commercial**
Intake, kg/cow/d	19.0	20.1	19.5	0.43	0.184	0.127	0.300
**Digestibility, g absorbed/kg ingested**
Dry matter	586c	643a	632b	2.50	< 0.001	< 0.001	0.005
Organic matter (OM)	627b	676a	670a	3.00	< 0.001	< 0.001	0.121
Crude protein (CP)	603b	650a	635a	5.70	< 0.001	< 0.001	0.069
Ether extract	653b	702a	696a	5.90	0.001	0.003	0.631
Non-structural carbohydrates	684b	732a	723a	4.30	< 0.001	< 0.001	0.147
Neutral detergent fiber	566c	637a	615b	5.20	< 0.001	< 0.001	0.006
Acid detergent fiber	511c	582a	559b	5.20	< 0.001	< 0.001	0.006
**Nutritive value**
TDN (g/kg DM)^b^	612c	666a	652b	2.70	< 0.001	< 0.001	0.001
DE (Mcal/kg DM)^b^	2.70c	2.94a	2.88b	0.01	< 0.001	< 0.001	0.009
ME (Mcal/kg DM)^b^	2.73c	2.97a	2.91b	0.01	< 0.001	< 0.001	0.002
NEL (Mcal/kg DM)^b^	1.38c	1.51a	1.48b	0.007	< 0.001	< 0.001	0.004
UFL (Mcal/kg DM)^c^	2.43c	2.66a	2.60b	0.01	< 0.001	< 0.001	0.007
Digestible OM intake, kg/cow/d	10.6b	12.1a	11.6a	0.35	0.004	0.002	0.345
TDN intake, kg/cow/d	11.6c	13.4a	12.7b	0.36	< 0.001	< 0.001	< 0.001
ME intake, Mcal/cow/d	51.9c	59.7a	56.7b	1.44	< 0.001	< 0.001	< 0.001
Digestible CP intake, kg/cow/d	1.81b	2.06a	1.95a	0.031	0.005	0.004	0.071

Means in the same row with different letters differ (P < 0.05); SEM, standard error of the mean.

^*a*^Diet: Control diet contained 30% berseem clover + 30% corn silage + 15% soybean meal + 25% yellow corn, without additives, or supplemented with *E. faecium* EGY_NRC1 (isolated to contains 1.1 × 10^9^ CFU/g) or commercial *E. faecium* NCIMB 11181 (isolated to contains 2 × 10^12^ CFU/g) at 2 g/kg feed daily/cow.

^*b*^TDN, total digestible nutrients; DE, Digestible energy; ME, Metabolizable energy; NEL, Net energy for lactation. All have been calculated according to NRC equation.

^*c*^UFL = unité fourragère du lait (net energy requirements for lactation equivalent of 1 kg of standard air-dry barley) calculated according to INRA equation.

Isolated or commercial *E. faecium* did not affect concentrations of serum total protein, albumin, globulin, albumin: globulin ratio and urea-N (Table 4). Both isolated and commercial *E. faecium* increased serum glucose (*P* = 0.002) and decreased serum cholesterol (*P* = 0.002). The commercial *E. faecium* decreased (*P* = 0.002) serum triglycerides and ALT (*P* = 0.038), while the isolated *E. faecium* decreased serum AST (*P* = 0.023).

**Table 4:** Experiment 2: Blood parameters of Holstein cows fed diets supplemented with isolated or commercial *E. faecium* as probiotics.

	**Diet** ^**a**^			**SEM**	***P*** **-value**		
	**Control**	**Isolated**	**Commercial**		**Diet**	**Control vs. others**	**Isolated vs. Commercial**
Total proteins, g/dL	9.58	9.93	9.96	0.15	0.193	0.073	0.897
Albumin, g/dL	5.35	5.60	5.52	0.14	0.480	0.252	0.715
Globulin, g/dL	4.23	4.33	4.43	0.13	0.589	0.385	0.590
Albumin: globulin ratio	1.29	1.32	1.27	0.06	0.833	0.943	0.553
Urea-N, mg/dL	78.9	76.8	81.3	1.54	0.144	0.917	0.052
Glucose, mg/dL	74.0b	81.5a	80.3a	1.39	0.002	0.004	0.533
Cholesterol, mg/dL	172a	152b	144b	5.20	0.002	0.007	0.313
Triglycerides, mg/dL	109a	101a	97b	1.30	0.002	0.002	0.034
AST, Units/L	35.3a	30.0b	33.0a	1.27	0.023	0.021	0.012
ALT, Units/L	23.5a	22.3a	20.6b	0.82	0.038	0.049	0.016

AST, Aspartate aminotransferase; ALT, Alanine aminotransferase. Means in the same row with different letters differ (P < 0.05); SEM, standard error of the mean.

^*a*^Diet: Control diet contained 30% berseem clover + 30% corn silage + 15% soybean meal + 25% yellow corn, without additives, or supplemented with *E. faecium* EGY_NRC1 (isolated to contains 1.1 × 10^9^ CFU/g) or commercial *E. faecium* NCIMB 11181 (isolated to contains 2 × 10^12^ CFU/g) at 2 g/kg feed daily/cow.

#### Milk production, milk composition, and milk fatty acids

Compared to the control, the isolated *E. faecium* followed by the commercial *E. faecium* increase (*P* < 0.001) daily milk production (actual, ECM and FCM), daily milk component yields and milk energy output (Table 5). Moreover, the isolated *E. faecium* followed by the commercial *E. faecium* improved feed efficiency compared to the control treatment. Treatments did not affect the concentrations of milk components.

**Table 5:** Experiment 2: Milk production and composition, and feed efficiency of Holstein cows fed diets supplemented with isolated or commercial *E. faecium* as probiotics.

	**Diet** ^**a**^			**SEM**	***P*** **-value**		
	**Control**	**Isolated**	**Commercial**		**Diet**	**Control vs. others**	**Isolated vs. Commercial**
**Production, kg/d**
Milk	30.6c	35.9a	34.2b	0.31	< 0.001	< 0.001	0.001
Energy corrected milk (ECM)	28.9c	35.0a	33.1b	0.42	< 0.001	< 0.001	0.002
4% Fat corrected milk (FCM)	28.5c	34.4a	32.5b	0.41	< 0.001	< 0.001	0.001
Total solids	3.76c	4.55a	4.33b	0.05	< 0.001	< 0.001	0.003
Solids non-fat	2.67c	3.21a	3.07b	0.03	< 0.001	< 0.001	0.006
Protein	1.01b	1.20a	1.15a	0.01	< 0.001	< 0.001	0.054
Fat	1.08c	1.34a	1.26b	0.02	< 0.001	< 0.001	0.006
Lactose	1.46c	1.76a	1.65b	0.02	< 0.001	< 0.001	0.001
Milk energy output, MJ/d	89.8c	109a	103b	1.32	< 0.001	< 0.001	0.001
**Composition, g/kg DM**
Total solids	122	126	126	2.8	0.153		0.941
Solids non-fat	87.1	89.1	89.6	1.56	0.085	0.001	0.570
Protein	32.7	33.2	33.6	0.28	0.114	0.037	0.316
Fat	35.2	37.1	36.7	0.99	0.102	0.053	0.598
Lactose	47.8	48.9	48.3	0.36	0.070	0.444	0.200
Milk energy content, MJ/kg DM	2.92	3.03	3.01	0.22	0.255	0.069	0.589
**Feed efficiency**
Milk: intake	1.61c	1.79a	1.75b	0.02	< 0.001	< 0.001	0.024
ECM: intake	1.52c	1.74a	1.70b	0.01	< 0.001	< 0.001	0.011
FCM: intake	1.50c	1.71a	1.67b	0.02	< 0.001	< 0.001	0.003

Means in the same row with different letters differ (P < 0.05); SEM, standard error of the mean.

^*a*^Diet: Control diet contained 30% berseem clover + 30% corn silage + 15% soybean meal + 25% yellow corn, without additives, or supplemented with *E. faecium* EGY_NRC1 (isolated to contains 1.1 × 10^9^ CFU/g) or commercial *E. faecium* NCIMB 11181 (isolated to contains 2 × 10^12^ CFU/g) at 2 g/kg feed daily/cow.

Both *E. faecium* strains decreased the proportion of C23:0 (*P* = 0.005) and increased (*P* = 0.017) C18:1 *trans*-9 in milk (Table 6). Compared to the control treatment, the isolated *E. faecium* increased (*P* < 0.05) the proportion of C18:1 *trans*-9, C18:2 *cis*-9-12 and C18:2 *trans*-10 *cis*-12, while the commercial *E. faecium* did not affect them.

**Table 6:** Experiment 2: Milk fatty acid profile (g/100 g fatty acids) of lactating Holstein cows fed diets supplemented with isolated or commercial *E. faecium* as probiotics.

	**Diet** ^**a**^				***P*** **-value**		
	**Control**	**Isolated**	**Commercial**	**SEM**	**Diet**	**Control vs. others**	**Isolated vs. Commercial**
C4:0	0.81	0.81	0.75	0.017	0.138	0.247	0.089
C6:0	0.97	0.95	0.99	0.016	0.391	0.876	0.206
C8:0	0.93	0.90	0.93	0.017	0.400	0.587	0.238
C10:0	2.68	2.74	2.75	0.128	0.907	0.686	0.960
C11:0	0.20	0.20	0.19	0.010	0.866	0.942	0.625
C12:0	3.33	3.38	3.35	0.193	0.983	0.892	0.920
C13:0	0.46	0.46	0.47	0.015	0.895	0.705	0.826
C14:0	12.3	12.2	12.3	0.180	0.829	0.709	0.664
C15:0	1.07	1.10	1.08	0.015	0.524	0.466	0.408
C16:0	34.4	34.2	33.9	0.240	0.502	0.343	0.536
C17:0	0.66	0.63	0.65	0.078	0.974	0.866	0.900
C18:0	9.36	9.20	9.21	9.205	0.817	0.559	0.974
C20:0	0.12	0.12	0.12	0.010	0.928	0.721	1.000
C22:0	0.05	0.05	0.05	0.002	0.936	0.737	1.000
C23:0	0.04a	0.03b	0.03b	0.002	0.005	0.023	0.610
C24:0	0.04	0.04	0.04	0.009	0.494	0.550	0.329
∑ saturated fatty acids (SFA)	67.4	66.9	66.8	0.480	0.698	0.531	0.868
C14:1*cis*-9	0.28	0.28	0.27	0.009	0.740	0.651	0.711
C14:1 *trans*-9	0.99	0.98	0.99	0.004	0.650	0.885	0.450
C16:1 *cis*-9	0.34	0.32	0.34	0.026	0.859	0.466	0.624
C16:1 *trans*-9	1.86	1.83	1.81	0.037	0.668	0.227	0.663
C18:1 *cis*-9	19.9	20.8	21.1	0.570	0.412	0.410	0.744
C18:1 *trans*-9	0.24b	0.26a	0.26a	0.007	0.017	0.037	1.000
C18:1 *trans* 11	0.97b	1.04a	1.00b	0.019	0.025	0.041	0.022
∑ monounsaturated fatty acids	24.5	25.5	25.7	0.570	0.405	0.505	0.781
C18:2 *trans*-9,12	1.76	1.76	1.72	0.084	0.911	0.938	0.730
C18:2 *cis*-9-12	0.17b	0.19a	0.18b	0.005	0.040	0.037	0.033
C18:2 *cis*-9 *trans*-11	0.40	0.41	0.40	0.024	0.986	0.405	0.892
C18:2 *trans*-10 *cis*-12	0.01b	0.02a	0.01b	0.002	0.029	0.036	0.033
C18:3 *trans*-9 *cis* 12,15	0.11	0.10	0.10	0.005	0.441	0.669	0.346
C18: 3 *cis*-9,12,15	0.40	0.39	0.40	0.026	0.954	0.829	0.806
C20:3 *cis*- 8,11,14	0.09	0.09	0.09	0.002	0.364	1.000	0.201
C20:4 *cis*-5,8,11,14	0.12	0.13	0.12	0.009	0.898	0.231	0.711
C20:5 *cis*- 5,8,11,14,17	0.03	0.03	0.03	0.006	0.604	0.442	0.353
C22:5, *cis*-7,10,13,16,19	0.17	0.16	0.16	0.004	0.354	0.265	0.450
∑ polyunsaturated fatty acids	3.25	3.26	3.20	0.075	0.818	0.220	0.587
∑ unsaturated fatty acids (UFA)	27.8	28.8	28.9	0.630	0.478	0.821	0.853
∑ conjugated linoleic acid^*b*^	0.41	0.42	0.41	0.024	0.969	0.882	0.860
UFA: SFA	0.41	0.43	0.43	0.012	0.493	0.276	0.848

Means in the same row with different letters differ (P < 0.05); SEM, standard error of the mean.

^*a*^Diet: Control diet contained 30% berseem clover + 30% corn silage + 15% soybean meal + 25% yellow corn, without additives, or supplemented with *E. faecium* EGY_NRC1 (isolated to contains 1.1 × 10^9^ CFU/g) or commercial *E. faecium* NCIMB 11181 (isolated to contains 2 × 10^12^ CFU/g) at 2 g/kg feed daily/cow.

^*b*^C18:2 cis-9 trans-11 + C18:2 trans-10 cis-12.

## Discussion

As shown in Figure 1, the MALDI-TOF MS showed that the isolated strain of *E. faecium* possess antimicrobial activity which was initially identified with a high confidence value of 2.31 that indicated a reliable identification of the isolate up to species level. This result agreed with that of 16S rDNA sequencing data (31). The phylogenetic analysis and the 16S rDNA sequencing assigned all the *E. faecium* EGY NRC1 isolates belonged to *E. faecium*.

### Experiment 1 (*in vitro* experiment)

The inclusion of isolated *E. faecium* (both strains) increased TGP. Generally, production of gases depends mainly on the composition and degradability of the incubated substrate and the concentration of the soluble components in the incubated substrates (32–34). In the present experiment, the composition of the diet and soluble components are the same between treatments indicating that the differences are mainly due to the strains of *E. faecium*. Jiao et al. (35) stated that specific LAB strains interact with rumen microorganisms to alter rumen fermentation with different modes of action in the rumen.

One promising area of research for the use of LAB in ruminant nutrition is its potential for reducing CH_4_ emissions (36). The isolated and commercial *E. faecium* decreased proportional CH_4_ and CH_4_ production per unit of degraded DM, OM, NDF and ADF which may be related to the reduced methanogenesis by stimulating the growth of lactate-utilizing bacteria such as *Selenomonas ruminantium, Megasphaera elsdenii*, and *Veillonella parvula*, which promotes H_2_ and CO_2_ sinks during the conversion of lactate to propionate (37). Moreover, LAB stimulates scavenging of hydrogen atoms to form propionate causing a lack of hydrogen as the main substrate for methanogenic bacteria (36). Cao et al. (11) observed a lowered *in vitro* CH_4_ production with LAB supplementation to a silage-based diet prepared with whole crop rice. To confirm our findings, further studies should consider analyzing rumen microbiome. The isolated strain of *E. faecium* increased *in vitro* TGP and decreased CH_4_ production compared to the commercial strain, with no clear reason indicating the need for experiments on genome sequence and their ability to produce bacteriocins and non-ribosomal synthesized peptides for explaining such effects (36). Such possible differences between strains or their metabolites will produce different abilities to shift rumen fermentation patterns, and to inhibit specific rumen bacteria that produce H_2_ or methyl-containing compounds that are the substrates for methanogenesis (36). Increasing TGP is not always advantageous but concurrent reduction in CH_4_ is definitely advantageous (38). The improved fiber digestion is the most probable reason for the lowered CH_4_ production (39).

The isolated and commercial strains increased dDM and dOM without affecting dNDF or dADF due to the high fermentative activities of LAB-probiotics. LAB can enhance the whole digestive process, the metabolic utilization of nutrients, and improve the feed efficiency by producing digestive enzymes (e.g., amylases, chitinases, lipases, phytases, proteases) or by just generating volatile fatty acids and B-vitamins: riboflavin, biotin, B_12_ vitamin (40). Cao et al. (11) observed increased *in vitro* DM degradability with LAB administration to total mixed ration silage containing whole crop rice.

The commercial *E. faecium* increased ruminal NH_3_-N concentration; however, the observed concentrations were greater than the optimum level (8.5 to over 30 mg NH_3_-N/dL) for rumen microbial proliferation (41). Basso et al. (10) observed no effects on ruminal pH when lambs were fed a diet treated with LAB. However, the isolated and commercial *E. faecium* decreased fermentation pH, which was somehow mirrored by the obtained SCFA. The isolated and commercial *E. faecium* increased SCFA concentrations, and this may be related to the improved DM and OM digestibility. So et al. (13) reported increased total SCFA in cows fed diets supplemented with LAB. The observed fermentation pH values in all treatments were greater than the optimum level (5.6) for ruminal fiber degrading and microbial growth (42), without changing ruminal fibrolytic and amylolytic microbial communities (43).

The quadratic effects of treatments (levels of *E. faecium*) on some parameters are important to emphasize the importance of defining the optimal dose of *E. faecium* that may improve animal performance. Therefore, the medium dose of *E. faecium* (2 g/kg feed) was recommended for the *in vivo* experiment on lactating cows, as this dose showed better effects compared to the low and high doses.

### Experiment 2 (lactation experiment)

It was not possible to obtain ruminal contents from cows because the experiment was done in a commercial farm without access to rumen-fistulated lactating cows. Therefore, the *in vitro* approach shows results that may partially explain and/or support the outcomes of the lactation experiment.

#### Feed intake, nutrient digestibility, and blood measurements

*E. faecium* supplementation did not affect feed intake which partly indicates unchanged feed palatability or acceptability. Other studies reported minor effects on feed intake in lambs and ewes fed with probiotics (8, 44), while So et al. (13) observed increased feed intake with LAB supplementation to lactating cows.

The isolated *E. faecium* improved the digestibility of DM, NDF and ADF compared to the commercial *E. faecium;* however, both isolated and commercial strains improved OM, CP, EE and NSC digestibility revealing the potential of *E. faecium* for improving nutrient digestibility. Similarly, So et al. (13) observed improved nutrient digestibility with LAB supplementation in lactating cows. Fiber degradability results are not consistent with those of the *in vitro* experiment (Experiment 1), which may be due to the conditions of both experiments (*in vitro* vs. *in vivo* conditions) and the fact that feeding bacterial direct fed microbial to livestock is based primarily on potential postruminal effects which is not available in the *in vitro* experiments (35). In this regard, probiotics change rumen fermentation rates and patterns (45), with beneficial effects on the gastrointestinal tract and rumen (46). Additionally, the supplement contains LAB which has a strong inhibitory effect on gastrointestinal infection by pathogens *via* the production of antimicrobial agents (46). It is expected that *E. faecium*, especially, the isolated strain, improved growth or activity of ruminal cellulolytic microbial populations and stabilizes the rumen pH (47), leading to improved nutrient digestion (45) and synthesis of microbial proteins (48). As previously noted, the isolated strain improved digestibility of DM, NDF, and ADF, and diet's nutritive value compared to the commercial strain, which confirm our assumption that the genome of both strains differs. The possible differed production of metabolites and bacteriocins may affect the composition of rumen microbiome, especially in those involved in fiber digestion (36). As observed in this study, previous experiments on lambs (49, 50), and lactating ewes (8) reported that probiotics improved nutrient digestibility.

All the measured serum biochemical parameters were within the standard physiological ranges for healthy cows (51). Treatments did not affect the concentrations of serum total protein, albumin, globulin, albumin: globulin ratio and urea-N indicating minimal effects on cow's nutritional status, muscle protein catabolism and kidney function (52). Both *E. faecium* strains increased serum glucose because of improved apparent OM and NSC. The levels of serum glucose were above the range (40–60 mg/dL), indicating an adequate energy supply for cows without risk of negative energy balance occurring (53). Further studies should follow on these findings as *E. faecium* supplementation could be helpful during the transition period.

Both *E. faecium* strains decreased serum cholesterol while commercial *E. faecium* decrease serum triglycerides showing the ability of *E. faecium* bacteria to deconjugate bile salts by a specific hydrolase causing a reduction in cholesterol and triglycerides absorption at the intestinal level (54). Additionally, the commercial *E. faecium* decreased serum ALT, while isolated *E. faecium* decreased serum AST showing its potential to improve liver activity, function, and health in cows (55).

#### Milk production, milk composition, and milk fatty acids

In this study, both isolated and commercial *E. faecium* increased daily milk production by 17.1 and 11.7%, ECM by 21.4 and 14.8% and FCM 20.9 and 14.2%, respectively which is similar to other studies (8, 13, 44, 56) that reported a positive relationship between supplementation of ruminant diets with probiotics and animal performance. The improved nutrient digestibility and increased blood glucose with probiotics supplementation can be considered the main reasons for increasing milk production (44). An improvement in digestibility and intake of nutrients (ME and TDN) supported release of important nutrients required for milk components synthesis (41). Moreover, the improved feed efficiency with the additives is another probable reason for the improved performance (41). As previously noted in Experiment 1, *E. faecium* decreased CH_4_ production indicating that a possible suppression in CH_4_ production would have redistributed energy for improved milk production (57). In this study, the antagonism of pathogenic organisms *via* antimicrobial effects, competition for adhesion sites or nutrients, stimulation of host defines mechanisms and inhibition of bacterial toxins can partially explain the improved milk production (9, 45).

Moreover, LAB increases the release of different endogenous substances, including antibacterial substances, nutrients, antioxidants, growth factors and coagulating agents, enhances performance and reduces the incidence of diarrhea by increasing the number of beneficial microorganisms in the rumen (45, 58) and enhancing animal health (59), which could also explain the increased milk production. As a result of unchanged feed intake and increased daily milk production, the isolated and commercial *E. faecium* improved feed efficiency by 11.2 and 8.7% (milk: intake ratio), 14.5 and 11.8 (ECM: intake ratio), and 14 and 11.3% (FCM: intake ratio), respectively. Frizzo et al. (60) observed that supplementing diets of lactating cows with LAB including *E. faecium* improved feed efficiency.

The weak effect of treatment on the concentrations of milk components is inconsistent with other experiments (8) that reported some changes in the concentrations of total n-3, n-6 fatty acids, polyunsaturated fatty acids and conjugated linoleic acids components when sheep were fed diet supplemented with LAB. However, more experiments are required to explore these effects.

Plasma uptake of fatty acids is responsible for about half of milk fatty acids and the rest amount is synthesized in the mammary gland (61, 62). The improved fiber digestion with the supplementation might be associated with altered milk fatty acid profiles. Both *E. faecium* strains increased the proportion of C18:1 *trans*-9, while the isolated *E. faecium* increased the proportion of C18:1 *trans*-9, C18:2 *cis*-9-12 and C18:2 *trans*-10 *cis*-12. The observed changes in milk fatty acids are a result of biohydrogenation of dietary PUFA (63). Further attention should be paid to the use of *E. faecium* on cow diets as they may increase the formation of bioactive fatty acids such as C18:1 *trans*-11 and C18:2 *cis*-9-12.

## Conclusion

Daily supplementation of cows with *E. faecium* (isolated and commercial) at 2 g/kg DM feed improved *in vitro* nutrient degradation and cows feed digestion, milk production and feed efficiency. The isolated strain of *E. faecium* showed better results compared to the commercial strain. Minimal effects were observed with *E. faecium* supplementation on milk fatty acid profile. Our data could be useful for producers looking for probiotics generated from byproducts for improving feed utilization and milk production.

## Data availability statement

The raw data supporting the conclusions of this article will be made available by the authors, without undue reservation. The names of the repository/repositories and accession number(s) can be found below: NCBI accession number MW856655.

## Ethics statement

The animal study was reviewed and approved by the Technical Committee of the Science, Technology & Innovation Funding Authority (STDF), Egypt (project STDF 33413).

## Author contributions

HA, AK, and HM: conceptualization, methodology, validation, formal analysis, visualization, and supervision. AK and EV-B-P: writing—original draft preparation and writing—review and editing. HA and AK: investigation and data curation. All authors have read and agreed to the published version of the manuscript.

## Conflict of interest

The authors declare that the research was conducted in the absence of any commercial or financial relationships that could be construed as a potential conflict of interest.

## Publisher's note

All claims expressed in this article are solely those of the authors and do not necessarily represent those of their affiliated organizations, or those of the publisher, the editors and the reviewers. Any product that may be evaluated in this article, or claim that may be made by its manufacturer, is not guaranteed or endorsed by the publisher.
